# Implementing Changing Talk Online Training: A Pragmatic Trial to Improve Dementia Care Communication in NHs

**DOI:** 10.1093/geroni/igaa057.1599

**Published:** 2020-12-16

**Authors:** Carissa Coleman, Kristine Williams, Yelena Perkhounkova, Maria Hein, Tim Beachy, Clarissa Shaw

**Affiliations:** 1 University of Kansas, Kansas City, Kansas, United States; 2 University of Kansas, Lawrence, Kansas, United States; 3 University of Iowa, Iowa City, Iowa, United States

## Abstract

The Changing Talk (CHAT) training effectively reduces elderspeak and subsequent behavioral challenges in residents with dementia in nursing homes. The Changing Talk: Online (CHATO) training was developed to increase staff access to education using a new online format. A pilot test was conducted to confirm the feasibility and effects of CHATO on training outcomes. In the initial nursing home, twenty-three direct care staff members in a small Midwestern nursing home enrolled in the course including 12 CNAs, 4 RNs, 2 LPNs, 2 CMAs, 1 Dietary Aide, 1 Social Worker, and 1 in Transportation. Two forms of a 13-item scenario-based test to measure knowledge gain were developed and tested. Of the 23 staff, 18 (78%) completed the post-test and 83% of completers achieved a post-test score of 70% or greater. Scores on the test improved from M=69% correct (SD=11.7) at pretest to 86% correct (SD=10.6) on posttest demonstrating knowledge gain (p=.024). Participants improved their recognition of elderspeak (21%) and person-centered communication (24%) in a video vignette and 86% self-reported improvement in their abilities to recognize ineffective communication and to apply more effective communication strategies in practice. A randomized control trial enrolled staff (N=187) in eight additional nursing homes. Preliminary results confirm improvements in test scores from M=70.6% correct (SD=15.8) at pretest to 77.2% correct (SD=14.1) on posttest and increased elderspeak recognition (p=.004). Relationships between nursing home characteristics, implementation strategies, and culture change measured by the Artifacts of Culture Change Tool and their relationship to communication outcomes will be presented.

